# How AI tools can—and cannot—help organizations become more ethical

**DOI:** 10.3389/frai.2023.1093712

**Published:** 2023-06-22

**Authors:** David De Cremer, Devesh Narayanan

**Affiliations:** Centre on AI Technology for Humankind, NUS Business School, National University of Singapore, Singapore, Singapore

**Keywords:** AI, ethical upskilling, moral agency, responsibility, ethical decision-making

## Abstract

In this paper, we argue that we cannot expect that AI systems—even given more data or better computational resources—will be more ethical than the humans who develop, deploy and use them. As such, we advocate that it is necessary to retain the responsibility for ethical decision-making in human hands. In reality, however, human decision-makers currently do not have the ethical maturity to meaningfully take on this responsibility. So, what to do? We develop the argument that to broaden and strengthen the ethical upskilling of our organizations and leaders, AI has a crucial role to play. Specifically, because AI is a mirror that reflects our biases and moral flaws back to us, decision-makers should look carefully into this mirror—taking advantage of the opportunities brought about by its scale, interpretability, and counterfactual modeling—to gain a deep understanding of the psychological underpinnings of our (un)ethical behaviors, and in turn, learn to consistently make ethical decisions. In discussing this proposal, we introduce a new collaborative paradigm between humans and AI that can help ethically upskill our organizations and leaders and thereby prepare them to responsibly navigate the impending digital future.

## 1. Introduction

As Artificial Intelligence^1^ (AI) systems are increasingly viewed as imperative for managing costs, increasing efficiencies, and raising productivity, organizations are increasingly adopting AI in a variety of decision-making contexts (Cockburn et al., 2018; Trunk et al., 2020; Dordevic, 2022). Organizational deployments of AI are especially noticeable in areas where they can enhance employees” ability to analyze and solve problems (e.g., Huang et al., 2019; Metcalf et al., 2019; Gregory et al., 2021). For example, AI is being adopted to facilitate human resource activities (Gee, 2017; Hoffman et al., 2018): such as recruitment and selection (Woods et al., 2020), structuring work schedules, making decisions for work teams, providing advice to those in authority positions (De Cremer, 2020; Parent-Rocheleau and Parker, 2022) and providing services to customers (Yam et al., 2021). Given recent advances in AI techniques—as notably seen in the tremendous performance improvements of large language models (LLMs) like GPT-3 and GPT-4—it is expected that the breadth and depth of organizational applications of AI will only continue to rise in the near future (cf. Hovy and Spruit, 2016; Felten et al., 2023). In turn, critical and morally sensitive decision-making tasks will increasingly be delegated to these intelligent technologies (Feuerriegel et al., 2022).

As we know, with great power also comes great responsibility. As organizations grow increasingly dependent on AI, a larger number of people will become vulnerable to AI-led decisions, and hence the more salient and urgent are the concerns about whether or not these AI systems will be ethical (De Cremer and Kasparov, 2021). That is, with increased dependence on AI, critical questions will arise about whether AI will harm the interests of the organization, be inclusive and respectful in treating its employees, and adhere to normative rules and generate fair decisions and outcomes (Lee et al., 2017; Kellogg et al., 2020).^2^ Given this situation, it is then also no surprise that calls for “ethical AI” and “responsible AI” have been gaining prominence in recent years (Anderson et al., 2018).

The emerging field of AI ethics is expansive in its scope—spanning a range of multidisciplinary perspectives on how to ensure that AI systems accord with norms of accountability, fairness, legality, transparency, and more. One aspect in this field that has received considerable attention—and which is the focus of the present paper—concerns the ethical consequences for the human parties that are on the receiving end of an artificial agent's recommendations. Research and policy-making efforts in this domain have been largely concerned with developing methods for ensuring that AI systems act fairly, identifying which party is accountable if anything goes wrong, and employing these systems in ways that their decisions are transparent and explainable to humans. Discussions of these key “principles” for ethical AI—i.e., fairness, accountability, transparency, etc.—are usually accompanied by various procedures and rules for ensuring compliance with these principles. However, as we will argue, these various principles and procedures will necessarily prove insufficient if our ultimate goal is to delegate ethical decision-making to AI systems. Ethical decision-making requires more than simply adhering to pre-defined rules and procedures: it also requires *moral agency*, robust *intentions* to act in moral ways even in unprecedented circumstances, and the ability to take *responsibility* for one's actions. As we will discuss, these requirements are why ethical decision-making ought to remain in human hands.

Even so, organizations seem increasingly willing to leave AI systems in charge of making morally-sensitive decisions. Our paper cautions against this tendency, and argues for the immutable role of humans in ethical decision-making. At the same time, this does not mean that AI has no role to play in helping organizations make more ethical decisions. Recognizing that human decision-making is itself fraught with biases and moral flaws, we propose a new collaborative paradigm in which AI systems can serve as an information tool for ethically upskilling human decision-makers, helping us decide and act morally in a more consistent manner. In so doing, we discuss how AI can—and cannot—help organizations become more ethical.

The rest of this paper proceeds as follows. In Section 2, we discuss the reasons why, despite a recent explosion of interest in issues of ethical AI, AI systems will remain fundamentally incapable of performing ethical decision-making in organizations. We then explore reasons why contemporary organizations seem to be ignoring this reality by adopting overinflated expectations about the moral properties of AI, and explicate the dangers of doing so. In turn, we argue that AI is best viewed as a “mirror”: one that reflects back to us the biases, discriminatory patterns and moral flaws that are deeply entrenched in our human cognition and social institutions. Subsequently, in Section 3, we elaborate on some ways in which human decision-making is itself routinely biased and flawed, and discuss the urgent need for “ethical upskilling”. In turn, we detail how AI systems—because of how they are trained to capture and emulate human behavior at scale—can play an invaluable role in these upskilling efforts. Finally, Section 4 concludes by discussing some implications of our view for organizations and society at large.

## 2. On the ethical properties of AI

Extant discussions on AI ethics have primarily progressed through the development and adoption of “principles”, “ethics codes”, “guidelines”, and “best-practice recommendations”, by policymakers, organizations and civil society groups alike. Government reports such as the European Union's “Ethical Guidelines for Trustworthy AI” (European Commission, 2019), and the Government of Singapore's “Model AI Framework” (PDPC, 2020), and industry reports on “Responsible AI” from Google (2022) and Microsoft (2022), are representative examples of this approach. Indeed, as Jobin et al. (2019) and Fjeld et al. (2020) demonstrate, there has been a recent explosion of documents outlining principles for AI ethics, and growing convergence in key principles—fairness, transparency, accountability, etc.—that are deemed to be fundamental for “ethical AI”.

These principles, however, have been subject to criticism in recent times. Hagendorff (2020), for instance, characterizes principles as “essentially weak”, since they are by design unenforceable by law, and hence cannot be meaningfully used to hold companies that deploy harmful AI systems to account. Related complaints about “ethics-washing”—i.e., using the language of principles in ambiguous and superficial ways to cover up wrongdoings, and “ethics-bashing”—i.e., reducing complex moral principles into simplistic “check-lists” and procedural rules—are increasingly commonplace (Bietti, 2020; Hao, 2020), and reflect growing skepticism about whether principle-led approaches are sufficient for ensuring that AI systems are deployed toward ethical ends.

In our view, however, the main problem with principle-led approaches cuts much deeper. Principles and guidelines are, in essence, fundamentally concerned with assessing whether decision-making *outcomes* can be seen as ethical or not. They focus mainly on the “what” of ethical decision-making, rather than the “how” or “why”: narrowing down our assessments of whether an AI system is “ethical” or not primarily in terms of whether their outcomes align with pre-defined rules and protocols. This reflects a “law of ethics” approach (Anscombe, 1958; Rességuier and Rodrigues, 2020): whereby our previous assessments of which situations are and aren't ethical are codified in terms of fixed rules that are meant to guide future actions.^3^ Contemporary approaches to “embed” ethics in AI systems—by incorporating existing ethical rules and principles as design constraints—are representative of this tendency (cf. Wallach and Allen, 2009; Conitzer et al., 2017; McLennan et al., 2020). However, crucially, to be an ethical *decision-maker* in an organization, it is not sufficient merely to simply optimize for good over bad outcomes.^4^

When we expect that AI systems will be ethical decision-makers in organizations, we are ultimately expecting that these systems would be able to reason, think and make judgment calls about the most morally appropriate course of action, across a wide range of managerial contexts (Mittelstadt, 2019; De Cremer, 2022). Crucially, given that organizations operate in a volatile, uncertain, complex, and ambiguous world, delegating ethical decision-making to AI systems means that we would need them to evaluate and respond to moral situations that they have not encountered previously, to determine the most appropriate, respectful, and moral decision at any given moment. To meet such expectations, AI systems would need to have a sense of *moral agency*, robust *intentions* to act in moral ways even in uncertain situations, and the ability to take *responsibility* for their actions (De Cremer and Moore, 2020; Hagendorff, 2020).

In our view, moral decision-making is an ability that requires decision-makers to understand what their moral responsibilities are, to extend care and awareness to others and their concerns, and to assess via an internal moral compass what is the most morally-appropriate course of action to take in unprecedented and constantly-evolving situations. Recent scholarly work has noted that ethics is a constantly renewed ability to “see the new” (Laugier and Chalier, 2013; Rességuier and Rodrigues, 2020), and in turn, to make culturally-sensitive and situationally-aware choices about which moral actions to take in any given context (De Cremer, 2022). This line of reasoning, in turn, implies that ethics is not an ability that intelligent machines could reasonably possess—clearly, it is fundamentally a human ability. Even though AI systems can be superior in executing certain well-defined tasks, by replicating what humans would do in similar situations, they are in essence clueless about the meaning and function of their task in its broader context. Moreover, crucially, their knowledge can often fail to generalize in *new* morally-significant situations (Mitchell, 2019). This entails that intelligent technologies are fundamentally limited when it comes to executing certain intelligent tasks that require quintessentially human traits—such as ethical judgments, symbolic reasoning, managing social situations, and creative ideation (Brynjolfsson and McAfee, 2014).

### 2.1. On misattributing moral agency to AI

Even though AI has no moral agency, why does it seem then that organizations seem so predisposed to attribute agentic properties to AI? In our experience, many organizational leaders seem increasingly persuaded by a common narrative propagated by “Big Tech” companies—that technology may well be the real (and only) solution to our social and ethical problems. This view reflects a mindset of “technosolutionism” (Morozov, 2013): a unilateral preference for technically-engineered solutions to complex social problems. Technology companies today seem to be increasingly popularizing this mindset. For example, the tech giant Google has been promoting “ethics-as-a-service”: a technology-first effort to help other organizations “navigate the tricky ethics of AI” (Simonite, 2020). In so doing, Google is inducing a mindset among business leaders that if intelligent technologies reveal biased decision-making outcomes, the solution is simply to *fix* the technology. As a result, business leaders increasingly think that ethics can now simply be delegated to intelligent machines, and therefore that they do not need to invest in becoming better and more responsible leaders themselves (De Cremer and Kasparov, 2021). In other words, thanks to AI, today's business leaders, with implicit endorsement from tech companies, are learning to think that it is easier to “fix” the biases of technologies than it is to retrain unethical humans to do the right thing (De Cremer and Narayanan, 2023).

It is crucial to critically appraise the growing influence of these technosolutionist narratives in the business world, particularly because they give rise to two important psychological phenomena that are likely to reinforce misplaced beliefs about the moral agency of AI, and in turn, make ethical decision-making in organizations much harder to achieve. These two phenomena are: (a) magical thinking about the humanlike properties of AI, and (b) increased confusion about who should take responsibility for AI-augmented decisions.

(a) Magical thinking: Technosolutionist narratives lead people to acquire overinflated beliefs about what AI systems can and cannot do (cf. Vicsek, 2020; Bareis and Katzenbach, 2021). In turn, this results in people reading agency, intentionality and humanlike intelligence into even the most mechanistic and programmed actions performed by AI systems. When we anthropomorphize AI systems in this way, we attribute to them magical—and humanlike—powers, and in so doing, come to believe that they can be *inherently* good or bad—like any other moral agent. As magical thinking about the powers of AI becomes increasingly commonplace, it seems increasingly socially acceptable to think that intelligent technologies could be held morally responsible for their actions and decisions.
(b) Confusion about responsibility-attribution: As people come to (mis)attribute humanlike and agential properties to AI, the notion that technological systems could be held morally responsible for the decisions made based on its output now starts to *seem* plausible. This can lead to deep confusion in real-world decision-making contexts, as those who work with AI systems to make decisions are no longer able to think clearly about who (i.e., workers or the AI system) should be held responsible for enacting various work-role responsibilities (see e.g., Hanson and Bar-Cohen, 2009; Brynjolfsson et al., 2018). As a result, AI systems may make employees feel more uncertain about the expectations associated with their work roles (e.g., Rizzo et al., 1970), and in turn, come to disengage from the moral ramifications of their work.

In our view, these beliefs emerging out of prevalent technosolutionist narratives are profoundly detached from reality. To understand why this is the case, however, it is necessary to take a closer look at how contemporary AI systems are developed, and what they are actually capable of.

### 2.2. A primer on contemporary AI systems, and what they can and cannot do

A full discussion of the technicalities of the machine learning (ML) techniques methods^5^ that underlie contemporary AI systems falls outside the scope of this paper. However, to better understand why the trends we discussed above (i.e., of attributing moral agency and responsibility to AI) are so deeply misplaced, it is important to have at least a broad understanding of what exactly contemporary AI systems are (and aren't) capable of. Our overall view is perhaps best summed up by Kate Crawford's famous slogan: “Most AI is neither artificial, nor intelligent” (Crawford, 2021).

In broad terms, ML models are “trained” by letting them analyze large and highly-dimensioned datasets to identify generalizable patterns—typically either in a “supervised” manner (i.e., with human-labeled examples of correct and incorrect inferences that the model learns to extrapolate patterns from), or in an “unsupervised” manner (i.e., where the model is given some sort of reward/cost function to optimize during its training). Even though engineers do not explicitly code formal inference rules or decision-making logics into their models, they still need to make numerous consequential decisions—to define the problem that needs solving, set the background conditions under which the model will be trained, define the reward/cost function that needs to be optimized, etc.—all of which entails considerable tinkering and experimentation (Agrawal et al., 2018). Moreover, as noted earlier, the value of contemporary AI systems is based firmly on the existence of “big data” (Brynjolfsson and McAfee, 2012; Varian, 2014). However, in most cases, the large datasets used for training and testing AI models are also indelibly shaped by human choices—particularly those of the “ghost workers” who label, clean, and verify data to make them usable for AI developers (Gray and Suri, 2019). Because of these significant human decisions that shape AI systems at every step of the way—from data-labeling, to training, to testing, to deployment—it seems like a misnomer to label these systems as truly “artificial”.

Even so, at first glance, these systems at least *appear* to be intelligent, in certain important ways. ML models can spot patterns in large and highly-dimensioned datasets that no human could reasonably parse, and base their predictions on complex patterns that would have been otherwise invisible to humans (cf. Burrell, 2016; Joque, 2022). Indeed, if humans were asked to parse comparably large amounts of complex data, our approach would inevitably resort to heuristic-based thinking, which would likely yield less useful patterns and insights compared to ML models (Korteling et al., 2018). Moreover, with more data and more powerful computational resources, AI systems can improve their performance over time, and learn to make even more accurate judgments about the situations that are represented in their training data. For these reasons, AI systems can sometimes be seen as viable replacements for human intelligence and intuition in certain use-contexts, because their self-learning capabilities can reliably mimic human behavior and actions by using accumulated data from behavioral observations and other such data sources (Russell and Norvig, 2016).

However, this “intelligence” has deep-seated limitations. ML techniques may reveal numerous explicit and implicit patterns in the datasets they are trained on—even patterns that humans would have been unable to see—but crucially, they do not reveal anything *new* that was not previously contained within this data (cf. Bender et al., 2021). This is an important limitation especially when it comes to expecting ethical decision-making from AI. As we have argued above, ethical decision-making requires the ability to deal with *new* situations—assessing what is morally appropriate, respectful, and socially acceptable in any given situation cannot be simply generalized from previous similar situations. Every ethical decision is a new decision—and even if trends from previous decisions might contain some clues about how to respond in a new decision-making context, they cannot fully determine what is morally appropriate in a new context.

Crucially, even the most sophisticated ML techniques cannot infer *meaning* from learning—as they lack the rich conceptual and emotional knowledge humans have about objects and experiences, it fails to draw analogies, and they are limited in understanding frames of references to engage in subtle contextual, symbolic and cultural interpretations of human language (Nath and Sahu, 2020; Mitchell, 2021; Ricci et al., 2021; Toews, 2021). As such, it is unreasonable to attribute intentionality, in any meaningful sense of the term, to AI. Indeed, intentionality requires that the agent has mental states and awareness that motivates them to set out to accomplish a goal to satisfy one's concerns, motives, and desires, which, as we explained earlier, are abilities AI does not have and therefore cannot be regarded as an agent acting with intent (De Cremer and Kasparov, 2022).

As we have argued, ethical decision-making is fundamentally dependent on having the right intentions, extending relational care to understand what potential outcomes might mean to those who are affected by these decisions, and taking responsibility for these decision outcomes. The fact that AI cannot be an ethical decision-maker is not due to its present-day technological limitations—i.e., it is not the case that some more data, or even some future technological breakthrough, will suddenly give AI systems the ability to make ethical decisions. Intentionality, care and responsibility are deeply human attributes, and as such, even the most sophisticated AI algorithms cannot truly “decide” on their own accord to do good or bad. Even when the outputs of certain AI systems result in good or bad outcomes, any assessment of the goodness or badness of these outcomes must ultimately come down to the intentions and preferences of the people and/or organizations that employ these systems.

### 2.3. AI as a “mirror”—reflecting human biases and moral flaws

If AI systems do not have moral agency and intentionality, how should we make sense of the fact that their outputs are often connected to important morally-significant outcomes? In our view, rather than thinking of AI as an ethical decision-maker in its own regard, it is more accurate to think of it as a *mirror*: one that reflects back to us the biases, discriminatory patterns and moral flaws that are deeply entrenched in our human cognition and social institutions. It is therefore inaccurate to assume that technologically sophisticated machines would be more ethical than us in the same way that, say, an ML-based chess engine could learn to find better chess moves than us. Rather, when AI systems are trained on large amounts of data about human behavior, they simply learn to capture more accurately the ways in which humans make decisions—biases and all. As such, large AI models can sometimes capture and reflect deep truths about how humans make moral decisions—even truths that might have otherwise remained opaque to us. Complaining about the biases and moral harms of AI, therefore, is like complaining about our image in the mirror!

Human biases and moral flaws can come to be embedded in AI systems throughout different stages of their conceptualization, development, deployment, and management.^6^ As our understanding of this fact—and more generally, of the biases of both humans and AI systems—has matured in recent times, there has been an explosion of technical research into carefully designing AI systems in ways that mitigate these biases and moral harms (cf. Silberg and Manyika, 2019; Guizzardi et al., 2020; Richardson and Gilbert, 2021). Despite this, however, the fact remains that AI systems cannot *transcend* the ethicality of their creators or the social context in which they were built. Technical solutions may allow us to reactively correct some of the biases that come to be embedded in AI models and datasets during their development, but as long as humans remain in charge of deploying and managing these AI systems, there remains a real possibility that the same biases could creep back in. Moreover, complaints about biased and unfair AI decisions have been steadily on the rise in recent times (cf. Silberg and Manyika, 2019; Ntoutsi et al., 2020)—suggesting that much work on these issues remains to be done.

What we are calling for, therefore, is a fundamental and much-needed shift in mindset. We should abandon our misplaced expectations that AI systems can be autonomous ethical decision-makers, and rather, we should remind ourselves of the importance and necessity of the moral compass of humans to guide us in making ethical decisions when intelligent machines are around. Crucially, if we would like to make decisions that are morally progressive compared to the ones we have made in the past, we cannot leave decision-making simply to AI. Recognizing the irreplaceable role of humans in ethical decision-making, in turn, points us to two urgent priorities. First, we need to invest more resources, time, and training in the art of “ethical upskilling”—to ensure that people use AI systems in responsible and socially-beneficial ways. Second, if AI is indeed a mirror that reflects our biases and moral flaws, we ought to take a long and careful look into this mirror: to uncover the discriminatory, unjust, and harmful patterns that are being reflected back to us, and in turn, to learn to become more and better ethical decision-makers.

## 3. On ethical upskilling and the role of AI

If the responsibility for ethical decision-making in general—and specifically to use powerful AI systems to achieve socially-beneficial ends—must be retained in human hands, the frequent and systematic biases, moral flaws and cognitive errors of humans become a matter of great concern. Of course, humans have been in charge of organizational decision-making long before the advent of AI, but our track record has been less than stellar: our organizations have long been beset by ethical scandals, crises of corporate governance, and irresponsible leadership (Adler, 2002; Knights and O'Leary, 2005). Indeed, as behavioral ethicists have shown, organizational actors face systematic cognitive limitations when it comes to choosing the right thing to do—this “bounded ethicality” can create a gap between our actual and intended behavior (cf. Tenbrunsel and Smith-Crowe, 2008; De Cremer et al., 2011; Chugh and Kern, 2016). Importantly, these biases and moral errors are often *implicit* and *unconscious*: their influence on our behavior can remain hidden and difficult to predict, and can sometimes be in direct opposition to our espoused beliefs and values (cf. De Cremer and Moore, 2020). As such, even the most well-intentioned person can sometimes decide to do bad things.

If our argument thus far is sound, it stands to reason that our unethical tendencies will likely be made *worse*—not better—with the advent of AI. If we continue to assume that AI systems will make ethical decisions on our behalf, and as a result, fail to step up and take responsibility for ensuring that AI-augmented decisions serve socially-beneficial goals, there is no reason to think that AI systems will magically figure out how to make ethical decisions by themselves. As De Cremer and Kasparov (2021) argue, for example, when people rely on technologies to make decisions, processes of “moral disengagement” can lead to them feeling less responsible for (im)moral decision outcomes, as they can perceive machines—rather than themselves—as being in charge of driving decision-making. Moreover, over time, if AI systems were left entirely in charge of ethical decision-making, there is a real danger of *moral deskilling* in humans: i.e., the prospect of becoming unduly reliant on technological assistance to the point where we might be unable to act morally on our own (cf. Vallor, 2015; Schwarz, 2019).^7^ In such cases, unethical decision-making in organizations is likely to be more frequent and severe. Moreover, bounded ethicality is known to place stricter constraints on ethical behavior in such situations of uncertainty and equivocality (cf. Sonenshein, 2007), and as such, people are likely to behave more unethically when dealing with AI—a relatively nascent technology whose long-term consequences on society are uncertain and difficult to evaluate. For these reasons, we argue, humans currently lack the ethical *maturity* to ensure that AI will be used for good.

Cultivating this much-needed ethical maturity, therefore, must be a key priority when we seek to prepare our organizations and business leaders for the impending digital future. We need to become better skilled at understanding our own good and bad behavior, and apply those insights to interventions and training sessions on how to use intelligent technologies in more responsible ways. Such awareness training of the *psychological underpinnings of (un)ethical behavior* can teach us when humans are most likely to show unethical behavior, and in turn, translate these learnings to influence the design and employment of intelligent technologies toward ethical ends. This is what we are calling “ethical upskilling”.

### 3.1. Using AI as a tool for ethical upskilling

The pedagogical and epistemological challenges of ethical upskilling cannot be overstated: how can we efficiently learn about, and teach organizations and business leaders to appreciate, the psychological underpinnings of their (un)ethical behavior? Crucially, given the fact that our biases and moral errors are typically a result of unconscious processes, merely pointing out our moral errors is often not enough: ethical training must raise awareness of *how* these errors come about, and provide pathways for students to manage their biases, adjust their behavior, and measure their progress (cf. Sparks and Pan, 2010; Emerson, 2017; Gino and Coffman, 2021). In our view, one way in which we can push ethical upskilling initiatives forward, and make them more relevant and insightful for decision-makers, is by harnessing the affordances of intelligent technologies. Even if AI systems cannot be ethical decision-makers in their own respect, they might at least play a role in teaching us how to be more ethical.

As we have argued above, AI systems capture, expose, and make prominent the biases and discriminatory patterns contained within the datasets they are trained on—and hence *mirror* our moral flaws back to us. This makes AI an excellent teaching tool to help humans identify our own ethical blindspots—as well as the ethical blindspots related to organizational culture, leadership, and our social institutions at large—and potentially pave the way for identifying strategies to overcome these blindspots. In other words, if decision-makers working with AI systems learn to take a critical look at the outputs of these systems, and think broadly and deeply about what these outputs mean in the broader organizational, social and political context, they would be in a good position to think carefully about what made similar previous decisions morally problematic, and how to avoid these in the future.^8^

For example, suppose an organization is using an AI system to aid in recruitment decisions, and has trained this system on historical data of its previous hiring decisions. This system, in turn, would become a reliable repository of the biases and harms perpetuated by the organization's previous hiring decisions—documenting, for example, previous instances of discrimination on gender, racial or religious grounds—and in turn, help decision-makers think more critically about how they might make more ethical decisions.^9^ Interacting with AI systems in this way entails treating their outputs *not* simply as recommendations of how to act in the future, but rather as ways of *learning* about how to avoid making mistakes from the past.^10^

In our view, three key aspects of contemporary AI make it especially useful as a tool for ethical upskilling:

(a) Scale: Because AI systems are typically trained on large and highly-dimensioned datasets, they have the potential to reflect discriminatory patterns and biases that would have otherwise remained invisible to human eyes (cf. Burrell, 2016; O'Neil, 2016). And, because AI systems are increasingly being deployed in far-reaching and consequential decision-making contexts, the risks of their decisions being biased and unethical are formidable, since this could potentially have an adverse effect on large groups of people. The massive scale of AI—both in its development and deployment—makes it so that they are increasingly not only *reflecting* our biases and flaws back to us, but *amplifying* them—making them easier to understand and harder to ignore. As such, the threat of powerful AI systems reproducing unethical human behavior at scale powerfully underscores the urgent need for humans in engage in ethical upskilling, and can in turn motivate us to take the risks of unethical AI seriously.
(b) Counterfactual modeling: ML models can be used to produce detailed and specific counterfactual scenarios—to see how small changes in inputs (say, a change in a given decision-subjects race, or a small increase in their income, etc.) can lead to different decision outcomes, and in turn, to different ethical consequences (cf. Chou et al., 2022). In our view, the learning opportunities offered by such counterfactual modeling techniques are profound. Counterfactuals could enable decision-makers to simulate the downstream effects of different possible decisions before committing to a particular choice, and in turn, gain a deep appreciation of the disparate impact of their decisions on different demographic groups (cf. Dai et al., 2022; Shang et al., 2022). Counterfactual explanations have also been used as an interpretability technique, to help users gain an intuitive appreciation for which factors are most important to an AI system when it makes its recommendations: which can similarly offer useful insights into the process of making ethical decisions (cf. Byrne, 2019; Keane and Smyth, 2020). Moreover, interacting with and thinking through counterfactual models may also inspire decision-makers to counterfactual thinking in their own decision-making process—which once again places them in good stead to carefully consider all relevant moral ramifications when making decisions (cf. Gollwitzer et al., 1990).
(c) Interpretability: If one were to ask a human who made a morally-significant decision *how* they came to this decision, their answers would likely be unsatisfactory. Human cognitive processes are, for the most part, opaque black-boxes—it is usually impossible to identify the full range of factors (both conscious and unconscious; internal and external) that lead us to decide and act in certain ways. As such, there is only so much that one could learn when analyzing another human's moral errors. When it comes to AI, however, there has been a recent explosion in research to make AI systems “interpretable”: to obtain high-fidelity, precise and easy-to-understand knowledge about the inner decision-making logic that these systems use to come to their decisions (cf. Mueller et al., 2019). Although there remain some critical shortcomings with making an AI system interpretable, decision-makers can sometimes have access to information about the key variables and logic that undergirds the system's decision-making process.

An AI model trained on data about a company's previous hiring decisions might, when made interpretable, reveal that these hiring decisions were unreasonably biased against certain racial or gender minorities. Or, an AI system used for performance management might, when made interpretable, reveal that certain groups of precarious low-paid workers are unreasonably surveilled more and pushed to work longer and harder than their better-paid white-collar colleagues. Or, a credit-scoring AI system that was explicitly designed to not consider racial categories when issuing credit scores might, when made interpretable, reveal that some other seemingly innocuous variable (e.g., postal codes) was being used as a proxy to racially profile credit-score recipients.^11^ In such ways, through a careful analysis of the factors that lead AI systems to make biased and unethical decisions, and critical reflection on why these factors might be systematically connected to unethicality, decision-makers can come to a more nuanced understanding of what is needed to avoid making unethical decisions.

However, for the moment, the usefulness of interpretability for ethical upskilling remains limited. First, as AI systems are becoming larger and more complex, it is becoming increasingly difficult to ensure that they remain meaningfully interpretable to humans (cf. Arrieta et al., 2020; Long, 2020). Second, even given rapid advances in interpretability techniques, there remains much work to be done in making sure that these techniques faithfully reveal the inner logics of AI systems, rather than simply providing misleading and over-simplified explanations (Ribeiro et al., 2016; DiMarco, 2021). Finally, the interpretation of an AI system's explanations is fundamentally subjective. It is unclear whether people will be able to draw meaningful and unambiguous ethical lessons by scrutinizing the content of an AI system's explanations (Ananny and Crawford, 2018; Rudin, 2019).

These limitations notwithstanding, in our view, it is still useful to keep the affordances of interpretable AI in mind as we think about how AI can serve as a tool in ethical upskilling. Interpretability techniques have seen rapid advances in their sophistication and effectiveness—and there are already a few cases where interpretability techniques can reveal morally useful knowledge about decision-making processes that might have otherwise remained opaque. While much work remains to be done to ensure that interpretable AI meaningfully serves ethical upskilling, it is important to note that interpretability should not be viewed as some sort of magic repository for moral knowledge, but rather as providing a starting point for critical conversations on how to consistently make ethical decisions.

As such, when we use AI as a tool for ethical upskilling, we appropriately acknowledge the powers and capabilities of these powerful technologies in helping us make more ethical decisions, while avoiding the mistake of thinking that AI systems can make such decisions by themselves. We should not throw the proverbial baby out with the bathwater: even if AI systems cannot make ethical decisions by themselves, they can still play a crucial role in helping us become more consistent ethical decision-makers.

## 4. Toward a new collaborative paradigm

We have argued that it is deeply unreasonable to expect that AI systems—even given more data or better computational resources—will be more ethical than the humans who develop, deploy and use them. This entails that the responsibility for ethical decision-making must remain in human hands. However, as we have discussed, it does not seem that human decision-makers currently have the ethical maturity to meaningfully take on this responsibility. There is an urgent need, therefore, to broaden and strengthen the ways in which we ethically upskill our organizations and leaders, and AI has a crucial role to play in such efforts. Specifically, because AI is a mirror that reflects our biases and moral flaws back to us, decision-makers should look carefully into this mirror—taking advantage of the opportunities brought about by scale, interpretability, and counterfactual modeling—to gain a deep understanding of the psychological underpinnings of our (un)ethical behaviors, and in turn, learn to consistently make ethical decisions. This is, in our view, a key strategy for improving current attempts to ethically upskill our organizations and leaders for the impending digital future.

Our suggested approach represents a new kind of collaborative paradigm between humans and machines when it comes to ethical decisions: where AI plays a *supportive* role in helping human decision-makers make more ethical and responsible decisions (cf. Table 1). In contemporary social and policy circles, there has been much discussion about the need for a “human-in-the-loop” for algorithmic decision-making: where the role of the human in decision-making is, at best, confined to scrutinizing, and in turn approving or rejecting, their AI system's recommended decision (see e.g., Zanzotto, 2019). In our view, however, a more accurate and responsible approach would be to have an “*AI-in-the-loop” for human decision-making*: where the role of AI is strictly confined to providing information about biases and moral flaws in potential outcomes, to help humans make better and more informed decisions.

**Table 1:** Key conceptual differences between the currently-dominant technosolutionist paradigm, and our proposed collaborative alternative.

	**Current technosolutionist paradigm**	**Our proposed collaborative paradigm**
Role of AI in ethical decision-making	Replacing humans as ethical decision-makers.	Serving as informational tools for ethically upskilling humans
Conceptualization of AI's moral attributes	AI as a moral agent, capable of transcending human morality.	AI as a “mirror”, reflecting our human biases and moral flaws back to us.
Conceptualization of what is needed for ethical decision-making	Ability to optimize for good over bad outcomes; to follow pre-defined rules and standards (i.e., the “law of ethics” approach).	Ability to respond to novel moral scenarios; to take responsibility for one's decisions; to intend to do the right thing.
Implications for human ethical decision-making	Humans grow increasingly dependant on AI for making ethical decisions, leading to moral disengagement (or worse, moral atrophy).	Humans ethically upskill, and over time, learn to make ethical decisions in a more consistent and robust manner.

Adopting this AI-in-the-loop approach has key implications for managers and organizational decision-makers. Crucially, managers must unlearn an increasingly common tendency to pass human responsibility and accountability over to algorithms: indeed, the phrase “the algorithm did it”, should be entirely purged from our vocabulary. Moreover, managers should be trained to be more aware of, and to be able to deal with, complex ethical business dilemmas—especially those pertaining to the use of intelligent technologies in organizations. Finally, managers need to be trained to recognize the human biases and flaws that underlie the decisions outputted by AI systems—and in turn, learn to recognize blindspots in their own thinking, as well as their organizational processes. As we have argued, we can learn to tap into the affordances of intelligent technologies to make these processes of ethical upskilling more instructive and valuable to decision-makers.

However, much more work remains to be done to implement this collaborative paradigm in organizational practice. While we hope to have provided a big-picture overview of what this paradigm might entail, we recognize that organizations may need further guidance on how exactly to incorporate AI into ethical upskilling initiatives. Even when we accept that AI is simply a mirror of human biases and moral flaws, we still need to learn how to carefully interpret and derive meaningful lessons from the image in this mirror! As such, implementing this collaborative paradigm is truly an interdisciplinary endeavor: we need technical research targeted at making AI systems better suited for ethical upskilling (especially in the domains of interpretability and counterfactual modeling), pedagogical research on how to educate business leaders to think critically and carefully about the moral implications of AI in decision-making, and organizational research on how exactly to include AI in organizational decision-making workflows (cf. Mittelstadt et al., 2016; Shrestha et al., 2019; De Cremer and Narayanan, 2023). We hope that the account sketched in this paper might provide guidance and impetus for future scholars to further help organizations pursue AI-augmented ethical upskilling initiatives.

In conclusion, as AI systems become increasingly ubiquitous in our organizations and societies, human decision-makers have an immutable and crucial role to play in ensuring that AI-augmented decisions are socially-beneficial and just. As we have argued, it is ultimately the moral compass of humans that we are relying on to guide the employment of these powerful technologies toward the social good. Without such a carefully-calibrated moral compass, both machines and humans would be at a loss.

## Author contributions

DDC came up with the idea. DDC and DN wrote the paper together. Both authors contributed to the article and approved the submitted version.

## Footnotes

^1^In this paper, we define AI broadly as computational systems that learn from large datasets to engage in pattern-recognition and problem-solving, which in turn promote the efficiency of organizations (Von Krogh, 2018): by making them more organized (Lwowski et al., 2017) and better able to achieve their objectives (Glikson and Woolley, 2020).

^2^Importantly, these questions and concerns long predate the advent of AI: even when organizational decision-making has been exclusively led by human decision-makers (as has been the case thus far), concerns about unfair biases when treating certain groups of employees over others, about systematic errors of judgment that lead to bad strategic decisions that harm the organization, and about unduly prioritising profit even to the detriment of society at large, to name a few, have been well-explored in business scholarship thus far (cf. Hammond et al., 1998; Campbell et al., 2009; Denning, 2019). These issues have perhaps taken a new form and urgency given the recent rise of AI, but their underlying concerns remain largely the same.

^3^Although moral philosophers sometimes distinguish between “ethics” and “morals” – typically using the former to discuss the appropriateness of actions, and the latter to discuss overarching principles and rules – in this essay we treat the two terms as largely interchangeable, unless explicitly specified otherwise.

^4^Here, it is important to note that we are *not* saying that embedded ethics approaches are unhelpful or otherwise unwarranted. Designing AI systems to better align with principles of fairness, accountability, trustworthiness, and other such normative principles has an important role to play in ensuring that AI-augmented decisions are ethical. Our argument is simply that these approaches will prove insufficient if the goal is to *delegate* organizational decision-making to AI. Put differently, AI systems can produce more or less ethical outcomes depending on how well they are designed, but they cannot be ethical *decision-makers* in their own regard. That said, we acknowledge that the status of AI as a moral agent in general has been up for debate. Various scholars have argued for AI systems to be more actively incorporated in ethical decision-making (cf. Moor, 2006; Behdadi and Munthe, 2020). However, as a recent review on this topic by Cervantes et al. (2020) notes, “there is a long way to go (from a technological perspective) before this type of artificial agent can replace human judgment in difficult, surprising or ambiguous moral situations” (p. 501). Although it falls outside the scope of this paper to substantively engage in proposals for artificial moral agency – especially outside the context of organizational decision-making – we would encourage interested readers to explore this topic further.

^5^The term “machine learning” is somewhat of a broad category - including both simpler techniques like regression and clustering, but also more powerful and complex neural networks. The subcategory of “deep learning” (including neural networks, generative models, etc.) are generally more architecturally complex – and a more technically-oriented discussion would do well to distinguish clearly between these various types of ML/DL models. For the purposes of this paper, however, we use the term “ML” as a catch-all, since our discussion does not hinge on any specific differences between types of ML/DL models.

^6^This is not to suggest that AI systems are only biased when humans *intentionally* develop, deploy, and/or manage these systems in biased and morally harmful ways. Our claim simply is that extant biases and moral flaws in AI are necessarily reflective of the biases and flaws of the humans and social forces that shape them. In other words, if an AI system is found to be biased, these biases may reliably be traced to biases and moral flaws in our human cognition and social institutions. The intentions (moral or otherwise) of those who develop, deploy, and manage these systems are, for the most part, irrelevant to our argument.

^7^Vallor (2015) and Schwarz (2019) discussions of moral deskilling are theoretically-grounded warnings of a somewhat speculative nature. However, more generally, the risks of deskilling in light of the advent of AI have been well documented and theorized for decades. The use of autonomous self-driving vehicles can adversely impact the competencies of human drivers (Bertrandias et al., 2021); the use of clinical decision-support systems can adversely impact the skills and experience of healthcare decision-makers (Mebrahtu et al., 2021); and the use of AI-generated artwork can adversely impact human creativity and artistic practices (Roose, 2022); to name a few. As such, insofar as we care about retaining our human ability to act morally even when faced with new situations, it is important to understand and counter the dangers of moral deskilling due to AI. Our thanks to an anonymous reviewer for making this important point.

^8^Interestingly, Kliegr et al. (2021) make a similar connection but in an opposite direction – arguing that our existing knowledge about the nature of human cognitive biases can prove instrumental in devising novel ways for debiasing AI systems. In other words, knowledge about human biases might be similarly important for” “ethically upskilling” AI systems. Our thanks to an anonymous reviewer for pointing out this connection.

^9^This is not merely a thought experiment: there have been several high-profile cases of AI-based hiring algorithms reproducing gender and racial biases in organizations, and as a result, there have been growing policy and scholarly efforts to urgently address these hiring biases (cf. Dastin, 2018; Raghavan et al., 2020).

^10^One interesting connection in this regard is to Clark and Chalmers' (1998) idea of an “extended mind'. Their proposal of *active externalism* – one where “the human organism is linked with an external entity [here, AI] in a two-way interaction, creating a coupled system that can be seen as a cognitive system in its own right” (p. 8) – can be seen as describing a human-AI collaborative paradigm that might plausibly be supported by the same arguments that we have made here (i.e., even if AI systems cannot be ethical decision-makers in their own regard, they can still play a role in improving human decision-making). There are, of course, important differences in terms of the phenomenology of human-AI interaction between their proposal (AI as part of the “extended mind' of a human decision-maker) and ours (AI as an information tool that reveals morally useful knowledge for human decision-makers to incorporate). That said, we acknowledge that one may plausibly extend the arguments in our paper to arrive at Clark and Chalmers' position, and we invite interested readers to pursue these connections further. Our thanks to an anonymous reviewer for bringing this to our attention.

^11^Although for the sake of narrative simplicity and readability, we have chosen to illustrate our point with examples of a somewhat hypothetical nature, it should be noted that there are a growing number of real-world examples of people learning morally useful things by scrutinizing AI systems. Amazon, notably, learned more about its historical record of hiring bias when an AI system trained on its previous employment records was unduly biased against women candidates (Dastin, 2018). Prince and Schwarcz (2020) offer a comprehensive review of the ways in which proxies mask discrimination in AI decision-making, and how these might be uncovered through more closely scrutinizing AI systems. In general, the interpretability literature is replete with examples of humans learning to improve their decision-making by examining the inner decision logic of AI systems – as well as the many shortcomings of interpretability that are yet to be fully addressed. Interested readers may refer to the comprehensive literature reviews of Doshi-Velez and Kim (2017) and Mueller et al. (2019), and/or to the theoretical expositions on this topic offered by Burrell (2016), Weller (2017), and (Narayanan, 2023) to name a few. Interested readers may also refer to Floridi et al. (2018) for a broader overview of the role of interpretability in ethical AI, and the applicability of ethical AI across a variety of domains including healthcare, environmental sustainability, and more.
